# TonEBP suppresses IL-10-mediated immunomodulation

**DOI:** 10.1038/srep25726

**Published:** 2016-05-10

**Authors:** Soo Youn Choi, Hwan Hee Lee, Jun Ho Lee, Byeong Jin Ye, Eun Jin Yoo, Hyun Je Kang, Gyu Won Jung, Seung Min An, Whaseon Lee-Kwon, Mario Chiong, Sergio Lavandero, Hyug Moo Kwon

**Affiliations:** 1 School of Life Sciences, Ulsan National Institute of Science and Technology, Ulsan, Republic of Korea; 2 Advanced Center for Chronic Disease (ACCDiS) & Center for Molecular Studies of the Cell, Facultad Ciencias Quimicasy Farmaceuticas & Facultad Medicina, Universidad de Chile, Santiago, Chile; 3 Department of Internal Medicine (Cardiology Division), University of Texas Southwestern Medical Center, Dallas, Texas, USA

## Abstract

TonEBP is a key transcriptional activator of M1 phenotype in macrophage, and its high expression is associated with many inflammatory diseases. During the progression of the inflammatory responses, the M1 to M2 phenotypic switch enables the dual role of macrophages in controlling the initiation and resolution of inflammation. Here we report that in human and mouse M1 macrophages TonEBP suppresses IL-10 expression and M2 phenotype. TonEBP knockdown promoted the transcription of the IL-10 gene by enhancing chromatin accessibility and Sp1 recruitment to its promoter. The enhanced expression of M2 genes by TonEBP knockdown was abrogated by antagonism of IL-10 by either neutralizing antibodies or siRNA-mediated silencing. In addition, pharmacological suppression of TonEBP leads to similar upregulation of IL-10 and M2 genes. Thus, TonEBP suppresses M2 phenotype via downregulation of the IL-10 in M1 macrophages.

Macrophages are an essential component of innate immunity as they play a central role both in the initiation and resolution of inflammation induced by pathogen or tissue damages^1^,^2^. Depending on the microenvironment, macrophages can acquire distinct functional phenotypes through undergoing different activation termed classical (M1) and alternative (M2)^3^,^4^. Classical M1 activation occurs in response to Toll-like receptor (TLR) ligands such as lipopolysaccharide (LPS) and interferon-γ (IFN-γ), and results in highly inflammatory macrophages by producing pro-inflammatory mediators^3^,^5^. In contrast, M2 macrophages are implicated in the resolution of inflammation, homeostatic maintenance and tissue remodeling and repair^3^,^4^. This cell type is more heterogeneous and is further classified into at least 3 subcategories - namely M2a, M2b, and M2c- that express different subsets of M2 marker genes and distinct functions^6^. M2a induced by interleukin (IL)-4 or IL-13 and M2b induced by combined exposure to immune complexes and agonists of TLRs exert immunoregulatory functions and drive type II responses, whereas M2c macrophages induced by IL-10 and glucocorticoids are more related to suppression of immune responses and tissue remodeling^6^,^7^. Plasticity and flexibility are key features of macrophages and of their activation states^6^,^8^. M1 and M2 macrophages promote the differentiation of neighboring cells to their common activation state and inhibit activation of the others. The same cells can, to some extent, be reversed from one to another functional phenotype. Moreover, the dynamic changes in macrophage phenotype frequently reveal divergent role of them in health and disease. Whereas M1 phenotype plays a causal role in inflammatory diseases such as rheumatoid arthritis, inflammatory bowel disease, and atherosclerosis, M2 or M2-like phenotype is associated with energy homeostasis and metabolic health beyond their role in resolution of pathologic inflammation^3^,^9^,^10^. Thus, the identification of molecules and mechanisms associated with phenotypic switch of them provides a molecular basis for macrophage-centered diagnostic and therapeutic strategies.

Tonicity-responsive enhancer binding protein (TonEBP), also known as nuclear factor of activated T cells 5 (NFAT5), belongs to the Rel family of transcriptional factors including nuclear factor κB (NFκB) and NFAT1-4^11^,^12^. TonEBP was initially identified as the central regulator of cellular response to hypertonic stress^11^,^13^,^14^,^15^. Recent studies have revealed that TonEBP is involved in the M1 activation of macrophages by promoting expression of pro-inflammatory genes in response to TLR4 activation^16^. Consequently, TonEBP haplo-defficiency is associated with reduced inflammation leading to prevention of inflammatory and autoimmune diseases including rheumatoid arthritis, atherosclerosis and encephalomyelitis, in mouse models^17^,^18^,^19^.

To explore the immunomodulatory function of TonEBP, we examined the role of TonEBP in the activation of M2 phenotype during M1 polarization of macrophages. We find that in M1-polarized macrophages TonEBP suppresses M2 phenotype via inhibition of IL-10 expression. Thus, TonEBP promotes M1 phenotype in two separate pathways: enhancement of M1 and suppression of M2.

## Results

### TonEBP suppresses M2 phenotype

**Figure 1:**
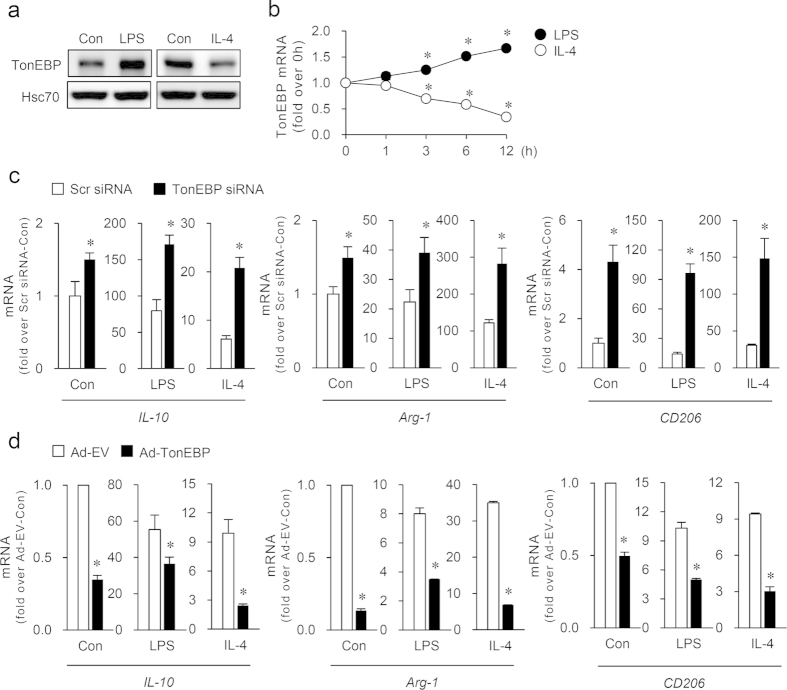
IL-4 diminishes the expression of TonEBP which reduces the expression of M2 genes in macrophages. (**a**) RAW264.7 cells were treated for 24 h with vehicle (Con), LPS (100 ng/ml), or IL-4 (10 ng/ml) and immunoblotted for TonEBP and Hsc70. (**b**) Cells were treated with LPS or IL-4 up to 12 h as indicated. Quantitative RT-PCR was performed for TonEBP mRNA and expressed in fold over 0h. SD bars are smaller than the circles (n = 3). *P < 0.05 compared to 0 h. (**c**) Cells transfected with scrambled (Scr) or TonEBP-targeted siRNA were treated for 6 h with vehicle (Con), LPS, or IL-4. Quantitative RT-PCR was performed for mRNA for IL-10, Arg-1, and CD206, and expressed in fold over Scr siRNA and Con. (**d**) Cells infected with adenovirus expressing TonEBP (Ad-TonEBP) or with empty vector (Ad-EV) were treated with LPS or IL-4. Data (mean + SD, n = 3) are representative of three independent experiments. *P < 0.05 compared to corresponding scrambled siRNA or Ad-EV.

Given the role of TonEBP in M1 gene expression and inflammatory diseases (see above), we explored the role of TonEBP in macrophage polarization in response M1 (LPS) and M2 stimuli (IL-4). While LPS increased TonEBP expression, as previously described^16^, we found that IL-4 reduced TonEBP expression (Fig. 1a) in mouse RAW264.7 macrophages. Time course experiments revealed that significant increase in TonEBP mRNA expression was reached in 3 h in response to LPS and the expression continued to rise to 12 h (Fig. 1b). In contrast, treatment with IL-4 caused significant and gradual reduction in TonEBP mRNA expression 3–12 h later (Fig. 1b). Thus, M2 signal reduced TonEBP expression while M1 signal promoted it.

During M1 polarization of macrophages in response to LPS, anti-inflammatory M2 genes including IL-10 are induced to provide a negative feedback^20^,^21^. We investigated whether TonEBP knockdown using siRNA-mediated gene silencing would influence the expression of M2 phenotype in M1 polarized macrophages. TonEBP was effectively knocked down by siRNA targeting TonEBP (Supplementary Fig. 1a). TonEBP knockdown enhanced mRNA expression of M2 genes such as IL-10, arginase-1 (Arg1), mannose receptor (CD206) and IL-4 receptor α (IL-4Rα) both in unstimulated and LPS-stimulated cells (Fig. 1c). On the other hand, induction of M1 genes iNOS and TNFα was reduced by TonEBP knockdown (Supplementary Fig. 1b), as previously reported^16^,^22^. Release of IL-4 and IL-13, inducers of M2 activation, was not affected by TonEBP knockdown (Supplementary Fig. 1c) demonstrating that TonEBP knockdown promoted M2 phenotype without changes in concentrations of IL-4 and IL-13 in M1-primed macrophages. We next asked whether TonEBP regulates expression of M2 genes in M2-polarized macrophages. TonEBP knockdown increased the expression of Arg-1, CD206 and IL-10 genes upon IL-4 stimulation (Fig. 1c). Reversely, TonEBP overexpression led to reduced mRNA expression of M2 genes in both M1 and M2 polarized macrophages (Fig. 1d), demonstrating that TonEBP directly suppresses M2 phenotype in both M1 and M2 macrophages.

### TonEBP suppresses IL-10 and its signaling in M1 macrophages

**Figure 2:**
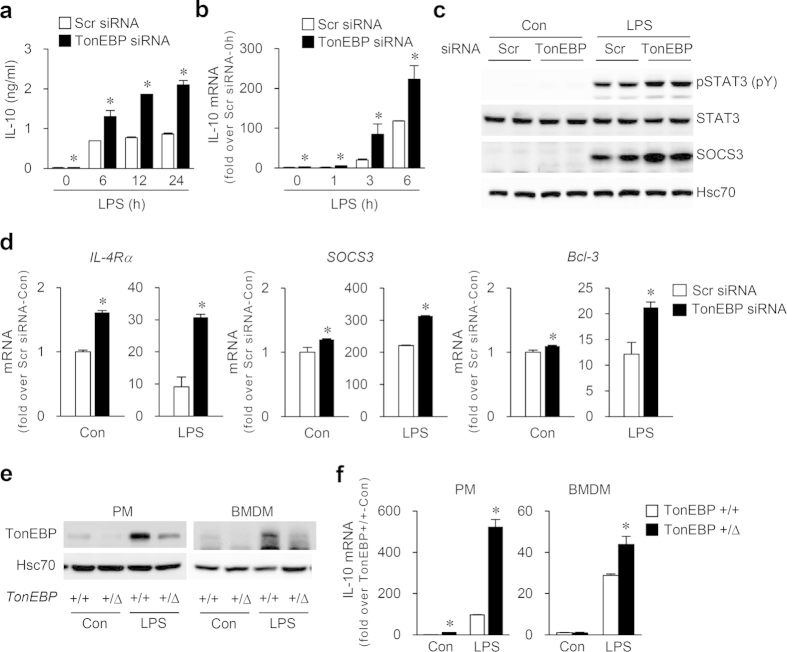
TonEBP reduces the expression and signaling of IL-10 in M1 macrophages. (**a**–**d**) RAW264.7 cells transfected with scrambled (Scr) or TonEBP-targeted siRNA and treated with LPS (100 ng/ml) for up to 24 h as indicated (**a,b**), 18 h (**c**) or 6 h (**d**). IL-10 concentration in the medium was measured by ELISA (**a**) and IL-10 mRNA abundance by quantitative RT-PCR (**b**). In (**c**), immunoblotting was performed for SOCS3, STAT3, phospho-STAT (p-STAT3), and Hsc70. In (**d**), mRNA for IL-4Rα, SOCS3, and Bcl-3 was measured. (**e,f**) Thioglycollate-elicited peritoneal macrophages (PM) or bone marrow-derived macrophages (BMDM) obtained from TonEBP^+/Δ^ mice and their TonEBP^+/+^ littermates were treated with vehicle (Con) or LPS (100 ng/ml) for 18 h (**e**) or 6 h (**f**). Immunoblotting for TonEBP and Hsc70 (**e**) and quantitative RT-PCR for IL10 mRNA (**f**) were performed. Data (mean + SD, n = 3) are representative of three independent experiments. *P < 0.05 compared to corresponding scrambled siRNA or TonEBP^+/Δ^ mice.

Among the M2 genes suppressed by TonEBP, we were interested in IL-10 because it is an essential anti-inflammatory cytokine, capable of reducing pro-inflammatory mediators and inducing M2 phenotype in macrophages^3^,^4^,^6^. We found that IL-10 secretion (Fig. 2a) and mRNA expression (Figs 1 and 2b) were higher in cells whose TonEBP was knocked down under basal conditions, and they increased steadily over time after stimulation with LPS. Because IL-10 leads to increase in expression of anti-inflammatory regulators such as suppressor of cytokine signaling-3 (SOCS3)^23^, IL-4Rα^24^ and Bcl-3^25^ through signal transducer and activator of transcription 3 (STAT3)^26^, we asked whether TonEBP affected the activation of STAT3 and expression of STAT3-inducible genes in RAW264.7 cells. In line with the increased secretion of IL-10, TonEBP knockdown augmented STAT3 phosphorylation in response to LPS (Fig. 2c) and enhanced LPS-induced expression of SOCS3 (Fig. 2c) and mRNA for IL-4Rα, SOCS3 and Bcl-3 (Fig. 2d). In order to confirm the regulation of IL-10 by TonEBP *in vivo*, we examined peritoneal macrophages (PM) and bone marrow derived macrophages (BMDM) obtained from the TonEBP^+/Δ^ mice displaying TonEBP haplodeficiency^15^. Both PM’s and BMDM’s from the TonEBP^+/Δ^ mice showed reduced TonEBP expression, both in unstimulated and LPS-stimulated conditions (Fig. 2e), and enhanced IL-10 mRNA expression compared to those from TonEBP^+/+^ littermates (Fig. 2f).

**Figure 3:**
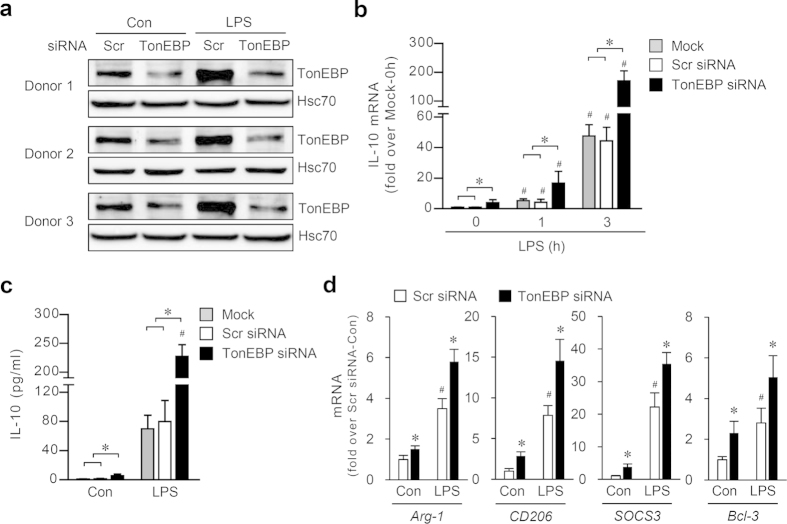
TonEBP reduces the expression of IL-10 in primary human macrophages obtained from 3 donors. The human macrophages were transfected with scrambled (Scr) or TonEBP-targeted siRNA. 48 h later, the cells were treated with vehicle (Con) or LPS (100 ng/ml) for 6 h (**a,d**), 1 and 3 h (**b**), or 6 h (**c**). Immunoblotting was performed for TonEBP and Hsc70 (**a**). In (**b**), IL-10 mRNA abundance was measured by quantitative RT-PCR. In (**c**), IL-10 concentration in the medium was measured by ELISA. In (**d**), mRNA for Arg-1, CD206, SOCS3, and Bcl-3 was measured. Mean + SD, n = 3. *P < 0.05 compared to corresponding scrambled siRNA.

We asked whether the suppression of IL-10 and its signaling by TonEBP was also present in human macrophages. To answer the question, we obtained primary monocytes from three donors. Macrophages differentiated from the monocytes responded to LPS by increasing the expression of TonEBP, IL-10, and anti-inflammatory regulators like RAW264.7 cells (Fig. 3). In addition, expression of the IL-10 and anti-inflammatory regulators was enhanced under basal and LPS-stimulated conditions in response to TonEBP knockdown. These data demonstrate that TonEBP suppresses IL-10 expression and M2 phenotype both in human and mouse macrophages.

### TonEBP suppresses the expression of M2 genes through IL-10

**Figure 4:**
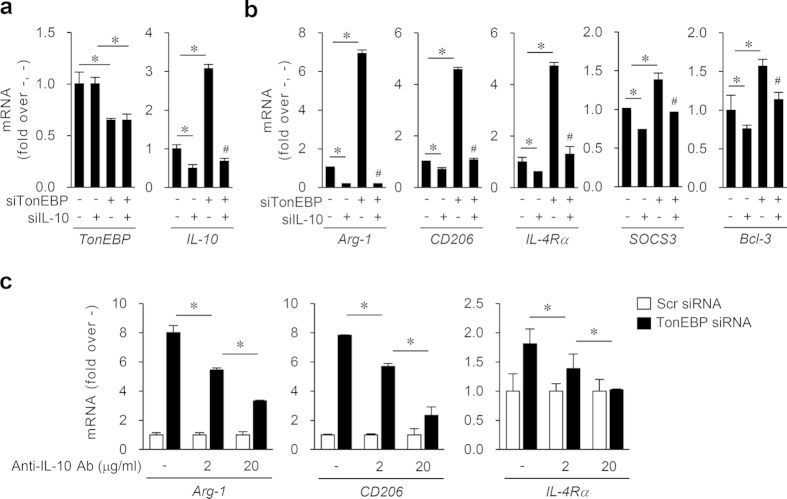
Reduction of M2 gene expression by TonEBP is dependent on IL- 10 in M1 macrophages. (**a,b**) Raw264.7 cells were transfected with scrambled (–), TonEBP-targeted, and IL-10-targeted siRNA in combinations indicated at the bottom. Total concentration of siRNA’s was equalized by adjusting the concentration of scrambled siRNA. The transfected cells were then treated for 6 h with LPS. mRNA expression for the genes indicated at the bottom was measured. (**c**) Cells transfected with Scr or TonEBP-targeted siRNA were treated for 6 h with LPS in the presence of anti-IL-10 neutralizing antibody (Anti-IL-10 Ab) at 2 or 20 μg/ml, or mouse IgG (−). Data (mean + SD, n = 3) are representative of three independent experiments. *P < 0.05.

We asked whether the enhanced expression of IL-10 in response to TonEBP knockdown was responsible for the expression of M2 phenotype. First, we examined the effects of exogenously added IL-10. We found that exogenous IL-10 addition promoted the expression of M2 genes Arg-1 and CD206 (Supplementary Fig. 2a) but inhibited TNFα expression (Supplementary Fig. 2b). In addition, the effects of the exogenous IL-10 and TonEBP knockdown were additive based on changes in Arg-1 and CD206 mRNA expression (Supplementary Fig. 2a) and TNFα mRNA expression (Supplementary Fig. 2b). Second, we examined the effects of silencing TonEBP and IL-10 using siRNA’s. The TonEBP-targeted siRNA silenced TonEBP without affecting IL-10, and the IL-10 targeted siRNA silenced IL-10 without affecting TonEBP (Fig. 4a). The mRNA levels of Arg-1, CD206, IL-4Rα, SOCS3 and Bcl-3 was reduced by IL-10 knockdown, while they were elevated by TonEBP knockdown (Fig. 4b). Notably, the enhancement of mRNA expression by TonEBP knockdown was almost completely abolished by TonEBP/IL-10 double knockdown (Fig. 4b). Third, we examined the effects of an IL-10 neutralizing antibody. The antibody prevented the enhanced expression of Arg1, CD206 and IL-4Rα by TonEBP knockdown (Fig. 4c). In aggregate, these data demonstrate that TonEBP suppresses M2 genes via down regulation of IL-10 in macrophages.

### TonEBP suppresses IL-10 transcription by blocking the recruitment of Sp1 to its promoter

**Figure 5:**
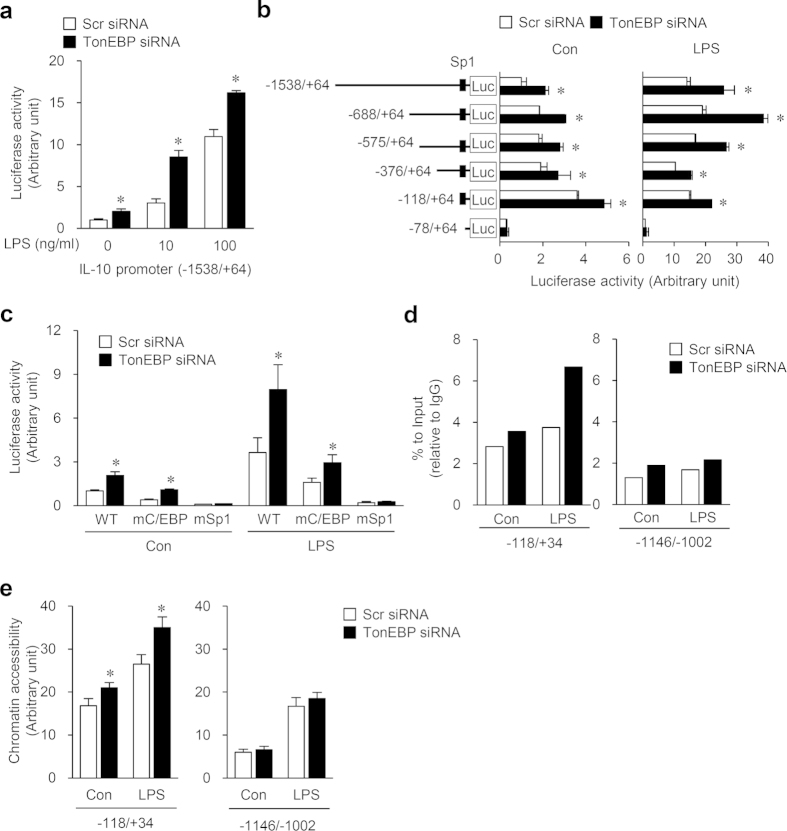
TonEBP suppresses the IL-10 promoter by blocking Sp1 recruitment and chromatin accessibility. RAW264.7 cells were transfected with scrambled (Scr) or TonEBP-targeted siRNA. (**a**) The siRNA transfected cells were transfected a second time with an IL-10 promoter-luciferase construct containing −1,538 to +64 region of the IL-10 promoter. Activity of luciferase was measured 8 h after treatment with 0, 10, or 100 ng/ml of LPS. (**b**) The siRNA transfected cells were transfected a second time with each of a series of 5′-deletion mutants of the IL-10 promoter construct. Activity of luciferase was measured 8 h after treatment with vehicle (Con) or 100 ng/ml LPS. A solid box labeled ‘Sp1’ denotes the Sp1 binding sequence. (**c**) The siRNA-transfected cells were transfected a second time with the −688/+64 promoter construct (WT), a mutant −688/+64 construct with its C/EBP site mutated (mC/EBP), or a mutant −688/+64 construct with its Sp1 site mutated (mSp1). Luciferase was measured after treatment with vehicle or LPS. (**d**) The siRNA transfected cells were treated for 1 h with vehicle (Con) or 100 ng/ml LPS. Chromatin immunoprecipitation was performed using anti-Sp1 antibody or IgG. Precipitated DNA along with input DNA was analyzed by q-PCR with primer pairs for two regions of the IL-10 promoter: a proximal region covering the Sp1 binding site (−118 to +34) and a distal region (−1146 to +1102). % to input value of Sp1 over that of IgG was calculated. A representative result from three independent experiments is shown. (**e**) The siRNA transfected cells were treated for 1 h with vehicle (Con) or LPS. Nuclei were prepared, and half of them were treated with micrococcal nuclease while the other half was treated without the enzyme. Genomic DNA was isolated and analyzed by q-PCR as above. Chromatin accessibility was calculated from (amount of PCR product in undigested sample)/(amount of PCR product in digested sample). Data (mean + SD, n = 3 to 5) are representative of four independent experiments. *P < 0.05 compared to corresponding scrambled siRNA.

We investigated molecular basis of TonEBP-mediated IL-10 gene suppression. First, we examined the M1 pathways that stimulate IL-10: secretion of IFN-γ and activation of ERK and p38^27^. Neither IFN-γ secretion nor activation of ERK and p38 secretion was affected by TonEBP knockdown (Supplementary Fig. 3) indicating that these pathways are not involved in the TonEBP suppression of IL-10. Next, we asked whether TonEBP suppressed the IL-10 promoter. We made an IL-10 promoter-luciferase reporter construct containing mouse IL-10 promoter sequence from nucleotides −1,538 to +64^28^. Macrophage cells transfected with the reporter construct expressed luciferase activity which was stimulated by TonEBP knockdown both in basal conditions and after stimulation with LPS (Fig. 5a). In order to define DNA sequence elements responsible for the stimulation, we made serial deletion constructs shown in Fig. 5b. Cells transfected with each of the constructs except for the smallest construct (−78/+64) displayed luciferase activity which was stimulated by TonEBP knockdown both in basal and LPS-stimulated conditions (Fig. 5b). Cells transfected with the −78/+64 construct showed a dramatically reduced luciferase activity which was not affected by TonEBP knockdown. Since this construct did not contain the binding site for Sp1 which is a key transcription factor for IL-10 induction in macropahges^28^, we decided to examine the role of Sp1. For this, we took the −688/+64 construct (WT) and made two mutant constructs from it using site-directed mutagenesis: mSp1 in which Sp1 binding site was disabled^28^ and mC/EBP in which C/EBP binding site was disabled^29^. TonEBP knockdown-dependent stimulation of luciferase was observed in cells transfected with WT and mC/EBP but not in those transfected with mSp1 (Fig. 5c) suggesting that Sp1 binding to the promoter is required for the stimulation of the IL-10 promoter by TonEBP knockdown.

A critical step in the activation of IL-10 promoter is the recruitment of Sp1 transcription factor to the proximal region of the promoter^30^. This is initiated by epigenetic transition in which phosphorylation of Histone H3 allows chromatin opening leading to the binding of Sp1 to its cognate DNA sequence. We asked whether TonEBP affected the binding of Sp1 to the IL-10 promoter using chromatin immunoprecipitation (ChIP) analyses. 1 h after stimulation with LPS, recruitment of Sp1 clearly increased in response to TonEBP knockdown in the −118/+34 region which contained the Sp1 binding site but not much in the −1146/−1002 region which was without a Sp1 binding site (Fig. 5d) suggesting that TonEBP suppressed Sp1 binding to the proximal region of the promoter, presumably DNA binding to the Sp1 binding site. Since TonEBP knockdown did not affect the protein expression and DNA binding affinity of Sp1 (Supplementary Fig. 4), we examined chromatin opening of the promoter region by analyzing sensitivity to micrococcal nuclease to calculate chromatin accessibility. Chromatin accessibility increased in response to TonEBP knockdown in the −118/+34 region but not in the −1146/−1002 region (Fig. 5e). Thus, TonEBP knockdown increased chromatin accessibility in the Sp1 binding site of the IL-10 promoter in correlation with increased Sp1 recruitment to the site.

### β-Lapachone, a chemotherapeutic agent, suppresses TonEBP expression and enhances IL-10 expression

**Figure 6:**
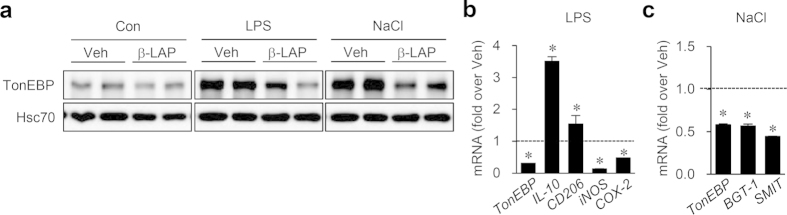
β-lapachone reduces TonEBP expression and enhances IL-10 expression. RAW264.7 cells were treated for 1 h with vehicle (Veh) or 10 μM β-lapachone (β-LAP), followed by a 6 h treatment with vehicle (Con), 100 ng/ml LPS, or additional 75 mM NaCl. (**a**) Immunoblotting was performed for TonEBP and Hsc70. (**b,c**) From the LPS and NaCl treated cells, mRNA for the genes indicated was measured by quantitative RT-PCR. mRNA abundance in β-LAP over that of Veh is shown. Data (mean + SD; n = 3 to 5) are representative of three independent experiments. *P < 0.05 compared to vehicle.

We screened a commercial library of natural compounds (BioMol, Plymouth Meeting, PA, USA) to find small molecules that suppressed TonEBP expression. Because TonEBP is essential for the expression of inducible nitric oxide synthase (iNOS) in LSP-stimulated macropahges^16^, we monitored LPS-dependent nitric oxide production using the Griess reaction^31^. Among the compounds that reduced the nitric oxide production, we found that β-lapachone, which has a variety of pharmacological effects including anti-inflammatory, anti-cancer and anti-angiogenic actions^32^,^33^,^34^, suppressed TonEBP expression both under LPS stimulation and hypertonicity (Fig. 6a). As expected from the reduced TonEBP expression, β-lapachone increased the mRNA expression of IL-10 and CD206 whereas suppressed the mRNA levels of COX-2 and iNOS in response to LPS (Fig. 6b). β-Lapachone also suppressed mRNA expression of tonicity-responsive TonEBP target genes such as sodium/chloride/betaine cotransporter (BGT-1)^35^ and sodium/myo-inositol cotransporter (SMIT)^36^ (Fig. 6c). The data provide strong evidence that the effects of β-lapachone on IL-10 expression is due to the reduction of TonEBP expression.

## Discussion

**Figure 7:**
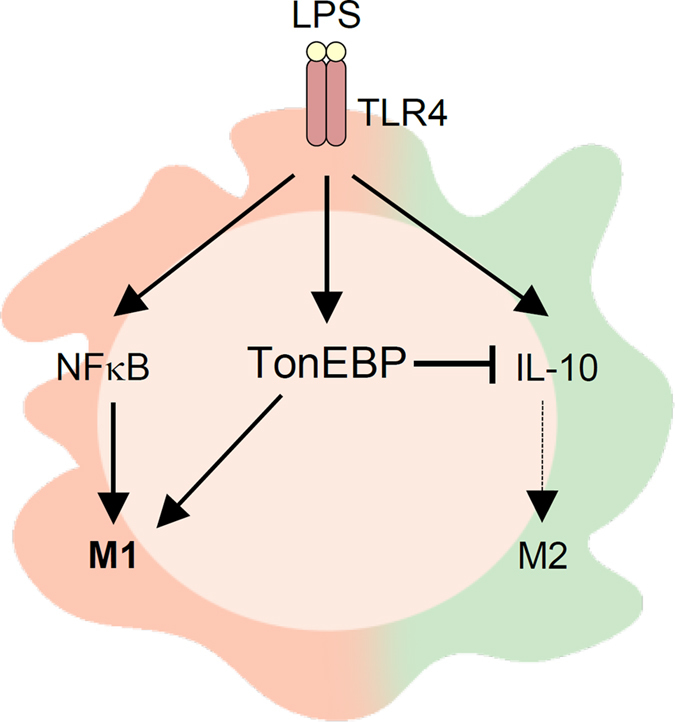
Schematic of proposed mechanism. In macrophages TLR4 engagement leads to stimulation of NFκB, which in turn promotes M1 gene expression. TLR4 engagement also increases TonEBP expression leading to stimulation of M1 gene expression. This study uncovers an additional action of TonEBP: suppression of the promoter of the IL-10 gene leading to decreased secretion of IL-10 and diminished M2 gene expression.

In macrophages TLR4 engagement induces the stimulation of NFκB and enhanced expression of TonEBP. Both NFκB and TonEBP bind to the promoters of M1 genes and stimulate their transcription leading to M1 activation^16^,^22^. The data presented here reveal that TonEBP has an additional activity. TonEBP suppresses M2 gene expression by way of reduced IL-10 expression (Fig. 7). TonEBP binds to proximal region of the IL-10 promoter and prevents chromatin access for Sp1 binding. Reduced secretion of IL-10 leads to diminished expression of M2 genes. Thus, TonEBP has a dual role in the macrophage polarization: stimulation of M1 plus suppression of M2.

The dual role of TonEBP suggests that moderate reduction of TonEBP could lead to effective anti-inflammatory status. Indeed, TonEBP haplo-deficient mice, which are normal in many aspects (unpublished observations), are effectively protected from inflammatory diseases. In a mouse model of rheumatoid arthritis, pannus formation is almost completely blocked^17^. In *ApoE*^*−/−*^ lines of mice fed with high fat diet, atherosclerotic lesions are reduced by 80% in a manner dependent on TonEBP haplo-deficiency in macrophages^18^. Renal inflammation is critical in the pathogenesis of diabetic nephropathy manifested by proteinuria and reduced renal function^37^. In patients with ~30 years of type I diabetes, those patients who display proteinuria has ~50% higher TonEBP activity in their monocytes compared to those patients without proteinuria^34^.

Unlike macrophage subset-specific cytokines such as TNFα and IL-4, IL-10 is produced by both M1 and M2 macrophages. IL-10 is a pleiotropic cytokine that controls inflammatory processes by suppressing the production of pro-inflammatory cytokines which are known to be transcriptionally controlled by NFκB^38^. IL-10 has a crucial role in infection by limiting the immune response to pathogens and thereby preventing inflammatory and autoimmune pathologies including rheumatoid arthritis, encephalomyelitis and atherosclerosis^39^,^40^,^41^,^42^,^43^. Furthermore, IL-10 promotes M2c macrophage polarization by inducing Arg-1, CD206, IL-4Rα, and others^23^,^24^,^25^,^26^,^44^. In this regard, the phenotype of TonEBP deficiency resembles IL-10-mediated immunomodulation.

Obesity-associated tissue inflammation is now recognized as a major cause of insulin resistance. IL-10 can inhibit the deleterious effects of pro-inflammatory cytokines on insulin signaling^45^. IL-10 overexpression in macrophages restrains diet-induced adipose tissue inflammation through the promotion of M2 phenotype^46^. Furthermore, muscle-specific overexpression of IL-10 increases whole-body insulin sensitivity^47^. Notably, loss of M2 macrophage exacerbates expression of inflammatory markers within the liver and adipose tissue^36^,^38^,^39^,^48^ and is not always compensated by inhibition of pathologic inflammatory phenotype alone^46^,^49^,^50^, demonstrating that the action of M2 macrophage is not limited to inhibition of pro-inflammatory molecules in tissue inflammation. Thus, suppression of M1 activation in combination with promotion of M2 activation is desirable as a therapeutic strategy against inflammatory diseases. As such, TonEBP should be an attractive target for obesity-associated insulin resistance and inflammation.

## Methods

### Isolation of the human primary monocytes and differentiation of monocyte-derived macrophages

The study was approved by the Institutional Review Board of the Ulsan National Institute of Science and Technology (UNISTIRB-15-25-A). Human blood was provided by the Korea Gynecologic Cancer Bank through Bio & Medical Technology Development Program of the MSIP, Korea. Mononuclear cells were isolated from heparinized blood using Histopaque-1077 (Sigma-Aldrich, St. Louis, MO, USA), according to the manufacturer’s instructions. Monocytes were further purified using CD14 microbead positive selection and MACS separation columns (Miltenyi Biotec, Bergisch, Germany), according to the manufacturer’s instructions. The macrophages were obtained after 7 days culture of human monocytes in RPMI-1640 medium supplemented with 10% FBS, 1% sodium pyruvate, 0.1% β-mercaptoethanol and human M-CSF (20 ng/ml; Miltenyi Biotec) following a previously published procedure^51^.

### Mice

All the methods involving live mice were carried out in accordance with the approved guidelines. All experimental protocols were approved by Institutional Animal Care and Use Committee of the Ulsan National Institute of Science and Technology (UNISTACUC-12-15-A). Mouse peritonitis was induced by intraperitoneal (i.p.) injection of 3% thioglycolate broth in 8- to 10-week-old mice as described previously^52^. Briefly, peritoneal cells were harvested at 72 hours after i.p. injection and macrophages were enriched by quick adhesion. BMDMs were differentiated with L929 conditioned medium as described previously^53^.

### Transfection and adenoviral infection

All siRNA duplexes were purchased from Integrated DNA Technologies (Coralville, IA, USA). Primary human macrophages and RAW264.7 cells were transfected with concentration-matched pairs of scrambled siRNA or siRNA against target genes using HiPerFect transfectant (Qiagen, Valencia, CA, USA) as previously described^51^ and lipofectamine 2000 (Invitrogen, Carlsbad, CA, USA) according to the manufacturer’s instructions, respectively. For overexpression, RAW264.7 cells were infected with the empty control virus (Ad-EV) or the adenovirus carrying the human TonEBP gene (Ad-TonEBP). A 1.6-kb fragment of the mouse IL-10 promoter (−1538/+64 pGL2B) and its 5′ deletion mutants were obtained from Addgene Inc. (Cambridge, MA, USA) and were subcloned into pGL3B (Promega, Madison, WI, USA). Sp1- or C/EBP-binding sites in −688/+64 promoter were mutated by a two-step PCR procedure using overlapping internal primers that contain a mutant sequence as published previously^29^. All plasmids were purified using an endotoxin-free purification system (Qiagen) and transfected using lipofectamine 2000 (Invitrogen).

### Western blot analysis and measurement of cytokine production

Western blotting was performed using standard methods. Cells were washed with cold PBS and lysed using RIPA buffer [10 mM Tris (pH 7.5), 150 mM NaCl, 1 mM EDTA, 1 mM EGTA, 1% Triton X-100] with 1 mM sodium orthovanadate, phosphatase inhibitor cocktail and protease inhibitor cocktail. Lysates were centrifuged at 14,000 rpm for 15 min at 4 °C. Protein were resolved by SDS-PAGE, transferred to nitrocellulose membrane (Whatman, Clifton, NJ, USA) and analyzed with anti-TonEBP^11^; Sp1 (Santacruz Biotechnology, Santa Cruz, CA, USA); pSTAT3, STAT3, SOCS3, p-ERK, ERK, p-p38, p38, p-AKT or AKT (Cell Signaling Technologies, Berkeley, CA, USA); anti-Hsc70 (Rockland, Gilbertsville, PA, USA). Cytokines were quantified by ELISA (R&D systems, Minneapolis, MN, USA) in culture supernatants.

### qRT-PCR

Total RNA from cultured cells was isolated using TRIzol reagent (Invitrogen). First-stranded cDNA was synthesized and subjected to quantitative real-time PCR using SYBR Green mastermix in a LightCycler 480 system (Roche, Rotkreuz, Switzerland). Gene expression was normalized to cyclophilin A using the change-in-threshold method. Primers used are described in Supplementary Table S1.

### Luciferase reporter assay

Cells were transfected for 48 h with scrambled or TonEBP-targeting siRNA for 48 h followed by transfection with the IL-10 promoter-driven luciferase reporter vector. The Renilla luciferase reporter plasmid was used as a control for transfection efficiency. 24 h post transfection, cells treated with LPS (100 ng/ml). After 8 h stimulation with LPS, cells were lysed in passive lysis buffer and luciferase assay was performed in the dual-luciferase reporter system (Promega).

### Chromatin immunoprecipitation (ChIP)

ChIP was performed using a commercial kit (Millipore, Bedford, MA, USA). In brief, cells were cross-linked with formaldehyde (1% final concentration; Sigma Aldrich) followed by addition of 125 mM glycine. After washing cells, chromatin fragmentation was done by sonication on ice to yield an average length of less than 500 bp. Supernatants of the fragmented lysates were diluted 10-fold with chromatin dilution buffer. Chromatin solutions were immunoprecipitated with anti-Sp1 or immunoglobulin G (IgG) antibody (Cell Signaling Technologies) at 4 °C overnight. After elution and reverse crosslinking the antibody/DNA complexes, DNA was purified by DNA purification kit (Qiagen) and analyzed by qPCR using primer pairs covering Sp1 binding region (−118 to +34) or distal region (−1146 to +1102) of the IL-10 promoter. Data were expressed fold over those obtained with genomic DNA (input) and IgG.

### Chromatin accessibility

Chromatin accessibility assay by qPCR was performed using MNase as previously described with minor modification^54^. Washed cells were lysed in cold NP-40 lysis buffer (10 mM Tris-HCl [pH 7.4], 10 mM NaCl, 3 mM MgCl_2_, 0.5% NP-40, 0.15 mM spermine, 0.5 mM spermidine, followed by incubation on ice for 5 min. Nuclei were pelleted by centrifuge at 5000 rpm for 3 min at 4 °C and resuspended with MNase digestion buffer without CaCl_2_ (10 mM Tris-HCl (pH 7.4), 15 mM NaCl, 60 mM KCl, 0.15 mM spermine, 0.5 mM spermidine). After centrifugation, the nuclei were resuspended with MNase digestion buffer supplemented with CaCl_2_. Then, half of the each sample was treated MNase with 5 unit/sample and the other half of that was treated digestion buffer, and samples were incubated at 37 °C for 1 min. The digestion reaction was stopped by addition of stop solution (100 mM EDTA/10 mM EGTA (pH 8.1) in 10 mM Tris-HCl [pH 7.4]). RNaseA (10 μg/sample) and proteinase K (100 μg/sample) were added and samples were incubated at 37 °C overnight. DNA, purified by phenol/chloroform/isoamyl alcohol extraction, was analyzed by qPCR using primer pairs covering Sp1 binding region (−118 to +34) or distal region (−1146 to +1102) of the IL-10 promoter. Chromatin accessibility was calculated as described in Fig. 5 legend (e).

### Statistical analysis

Data are presented as means + SD. Statistical significance (*p* < 0.05) was estimated by one-way analysis of variation with the *post hoc* Tukey’s test and two-tailed student’s t-test. All statistics was performed with GraphPad Prism 5.0 software (GraphPad, San Diego, CA, USA).

## Additional Information

**How to cite this article**: Choi, S. Y. *et al.* TonEBP suppresses IL-10-mediated immunomodulation. *Sci. Rep.* **6**, 25726; doi: 10.1038/srep25726 (2016).

## Supplementary Material

Supplementary Information
