# Supplementary material for: TonEBP suppresses IL-10-mediated immunomodulation
Source: Sci Rep. 2016 May 10;6:25726. doi: 10.1038/srep25726 (PMC4861964; doi:10.1038/srep25726)
Supplement: Supplementary Information [file srep25726-s1.pdf]

Supplementary Information

TonEBP suppresses IL-10-mediated immunomodulation

Soo Youn Choi[*], Hwan Hee Lee[*], Jun Ho Lee, Byeong Jin Ye, Eun Jin Yoo, Hyun Je Kang, Gyu Won Jung, Seung Min An, Whaseon Lee-Kwon, Mario Chiong1, Sergio Lavandero1,2, and Hyug Moo Kwon[‡]

School of Life Sciences, Ulsan National Institute of Science and Technology, Ulsan, Republic of Korea, 1Advanced Center for Chronic Disease (ACCDiS) & Center for Molecular Studies of the Cell, Facultad Ciencias Quimicasy Farmaceuticas & Facultad Medicina, Universidad de Chile, Santiago, Chile, 2Department of Internal Medicine (Cardiology Division), University of Texas Southwestern Medical Center, Dallas, Texas, USA

[*] Both authors contributed equally to this work.

Supplementary Methods

DNA affinity precipitation assay - Cells were washed with PBS and lysed in 20 mM Tris, pH 7.5, 150 mM NaCl, 1% Triton X-100, protease inhibitor, 1 mM sodium orthovanadate, and phosphatase inhibitor cocktail for 30 min at 4˚C. 0.5 mg/ml of protein was incubated with 30 nM biotinylated oligonucleotide probe overnight at 4°C in 7.2 mM Tris [pH7.5], 16 mM HEPES [pH 7.5], 4% glycerol, 166 mM NaCl, 0.4 mM EDTA, 0.8 mM $MgCl_2$, and 0.28% Triton X-100. Protein-DNA complexes were isolated using pre-cleared streptavidin-agarose bead and analyzed by western blotting.

Supplementary Figures

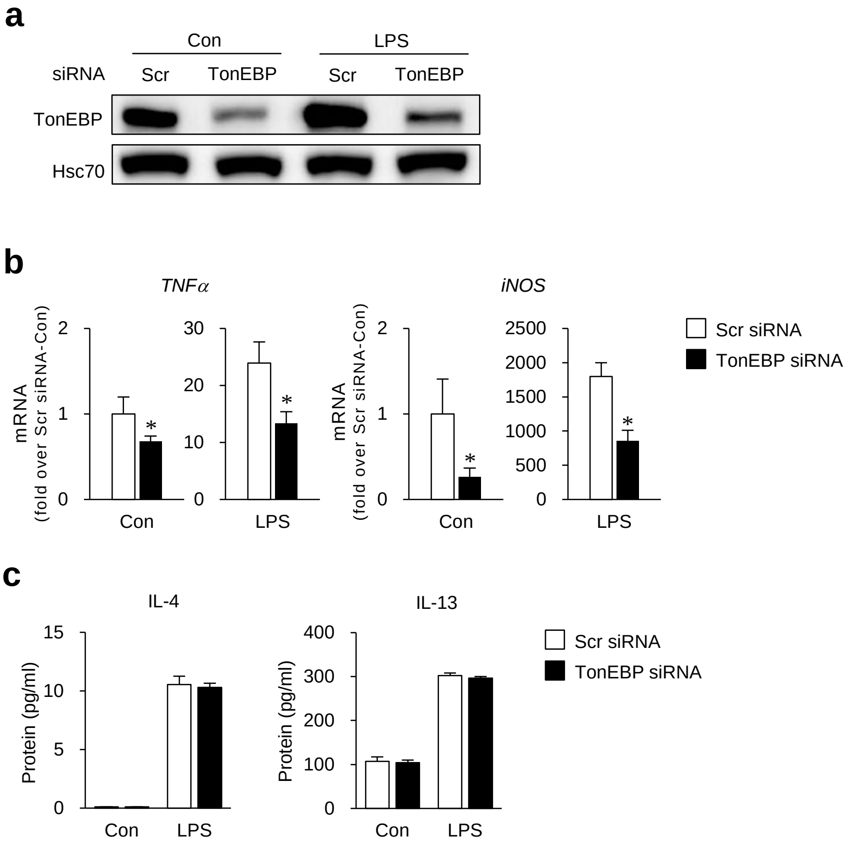

Supplementary Figure S1 (related to Figure 1). TonEBP enhances M1 gene expression in macrophages.

RAW264.7 cells were transfected with scrambled (Scr) or TonEBP-targeted siRNA followed by treatment with vehicle (Con) or 100 ng/ml LPS for 18 h (a) or 6 h (b, c). (a) Immunoblotting was performed for TonEBP and Hsc70. (b) Quantitative RT-PCR was performed for mRNA for TNFα and iNOS. (c) IL-4 and IL-13 was quantified in cell culture media using ELISA. Mean + SD, n = 3. * $P < 0.05$ compared to corresponding scrambled siRNA.

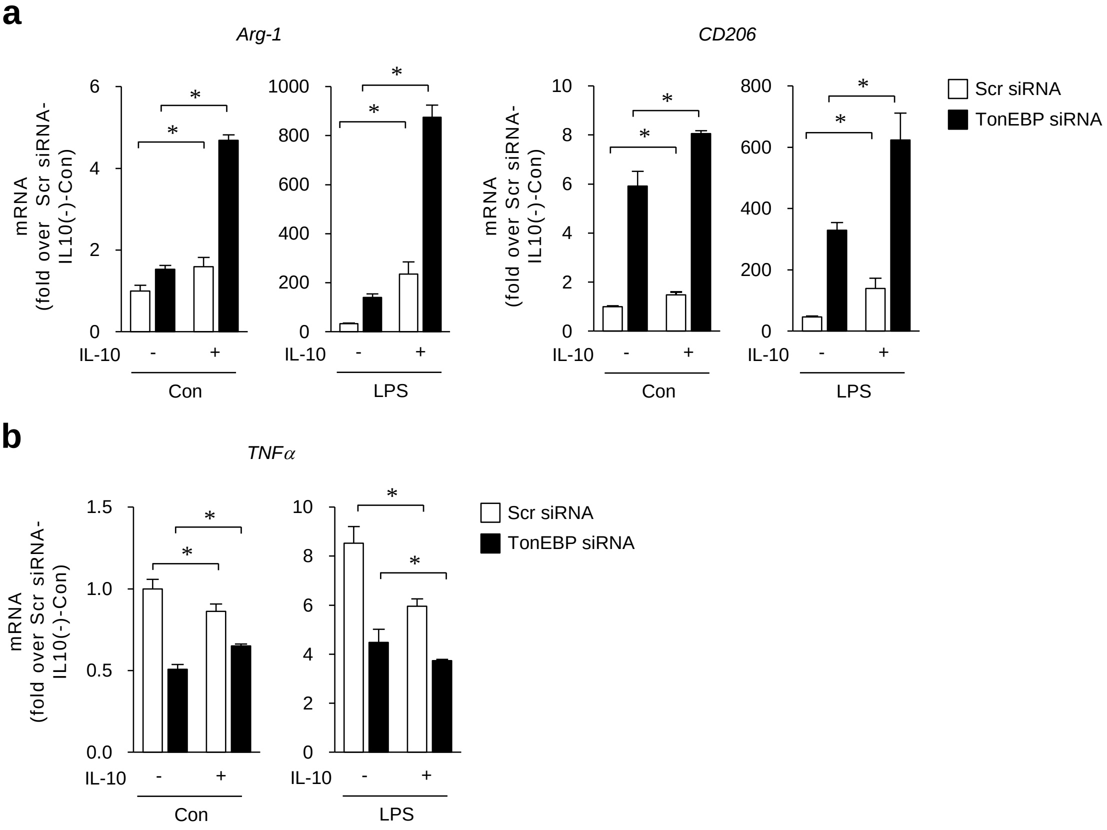

Supplementary Figure S2 (related to Figure 4). IL-10 enhances the effects of TonEBP knockdown on expression of M1 and M2 genes.

RAW264.7 cells were transfected with scrambled (Scr) or TonEBP-targeted siRNA followed by treatment for 6 h with vehicle (Con) or 100 ng/ml LPS in the presence or absence of murine IL-10 (1 ng/ml). mRNA for Arg-1, CD206 and TNFα were measured using quantitative RT-PCR. Mean + SD, n = 3. * $P < 0.001$.

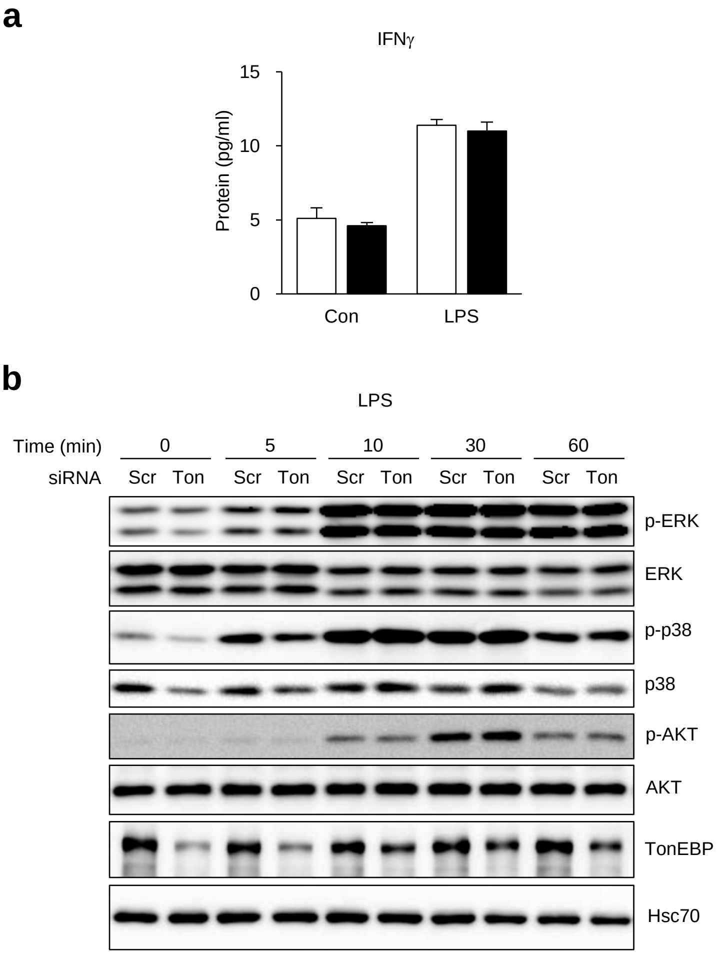

Supplementary Figure S3 (related to Figure 5). TonEBP knockdown does not affect IFN-γ secretion and activation of ERK and p38 MAPK.

RAW264.7 cells were transfected with scrambled (Scr) or TonEBP-targeted (Ton) siRNA followed by treatment with vehicle (Con) or LPS for 6 h (a) or up to 60 min as indicated (b). (a) IFN-γ in cell culture media was quantified by ELISA. Open bars – Scr siRNA; solid bars – TonEBP siRNA. (b) Total protein extracts were subjected to immunoblotting for phosphor-ERK (p-ERK), ERK, phosphor-p38 (p-p38), p38, phosphor-AKT (p-AKT), AKT, TonEBP, and Hsc70. Data shown are a representative result from three independent experiments.

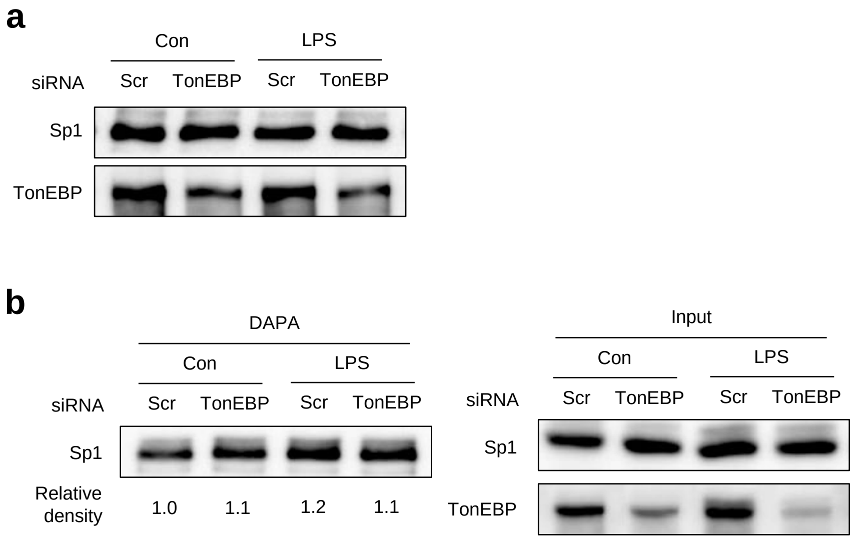

Supplementary Figure S4 (related to Figure 5). TonEBP knockdown does not affect the expression and DNA-binding of Sp1.

RAW264.7 cells were transfected with scrambled (Scr) or TonEBP-targeted siRNA followed by treatment with vehicle (Con) or LPS for 1 h. (a) Total protein extracts were subjected to immunoblotting for Sp1 and TonEBP. (b) Binding of Sp1 on 5'-biotinylated wild-type Sp1 binding motif was performed by DNA affinity precipitation assay (DAPA) using cell lysates. DAPA samples and cell lysates (input) were immunoblotted for Sp1 and TonEBP as indicated. Data shown are a representative result from three independent experiments.

Supplementary Table

Supplementary Table S1.

Gene	Forward primer	Reverse primer
Mouse *TonEBP*	5’-AAGCAGCCACCACCAAACATGA-3’	5’-AAATTGCATGGGCTGCTGCT-3’
Mouse *IL-10*	5’-ACCTGGTAGAAGTGATGCCCCAGGCA-3’	5’-CTATGCAGTTGATGAAGATGTCAAA-3’
Mouse *Arginase-1*	5’-CTCCAAGCCAAAGTCCTTAGAG-3’	5’-AGGAGCTGTCATTAGGGACATC-3’
Mouse *CD206*	5’-TCTTTTACGAGAAGTTGGGGTCAG-3’	5’-ATCATTCCGTTCACCAGAGGG-3’
Mouse *TNF*α	5’-TGGGACAGTGACCTGGACTGT-3’	5’-TTCGGAAAGCCCATTTGAGT-3’
Mouse *iNOS*	5’-GCTCATGCGGCCTCCTTT-3’	5’-CCTGTACGGGCATTGCT-3’
Mouse *IL-4R*α	5’-TGACCTACAAGGAACCCAGGC-3’	5’-GAACAGGCAAAACAACGGGAT-3’
Mouse *SOCS3*	5’-AGCTCCAAAAGCGAGTACCA-3’	5’-TGACGCTCAACGTGAAGAAG-3’
Mouse *Bcl-3*	5’-GACCTGGAGGTTCGCAATTA-3’	5’-CACCATGTTCAGGCTGTTGT-3’
Mouse *BGT-1*	5’-TTCGTGGCTGGGTTTGTTGT-3’	5’-ACAGCTGGGACAAAGGCATCAT-3’
Mouse *SMIT*	5’-ATTGCTCATGCCAAAGGCTCCA-3’	5’-ACTTGCATGCAGTGTTCTGGGT-3’
Mouse *Cyclophilin A*	5’-CTGCTGTCTTTGGAACTTTGTCTG-3’	5’-CAGCCATGGTCAACCCCACCG-3’
Human *TonEBP*	5’-AAGCAGCCACCACCAAACATGA-3’	5’-AAATTGCATGGGCTGCTGCT-3’
Human *IL-10*	5’-TTTCCCTGACCTCCCTCTAA-3’	5’CGAGACACTGGAAGGTGAATTA-3’
Human *Arginase-1*	5’-CAGGGCTACTCTCAGGATTAGA-3’	5’-CCGAAACAAGCCAAGGTTATTG-3’
Human *CD206*	5’-GGACGTGGCTGTGGATAAAT-3’	5’-ACCCAGAAGACGCATGTAAAG-3’
Human *SOCS3*	5’-TGGAATGTGTTGGAGGGAAG-3’	5’-GGAAGCTGAGGAATTGAAGGA-3’
Human *Bcl-3*	5’-GGTCAACCCTTCTGGAAACT-3’	5’-TGTATGGGTAGGTGCATGTG-3’
Human *Cyclophilin A*	5’-TTCATCTGCACTGCCAAGAC-3’	5’-TCGAGTTGTCCACAGTCAGC-3’
